# Detection of Renal Tissue and Urinary Tract Proteins in the Human Urine after Space Flight

**DOI:** 10.1371/journal.pone.0071652

**Published:** 2013-08-13

**Authors:** Lyudmila Kh. Pastushkova, Kirill S. Kireev, Alexey S. Kononikhin, Evgeny S. Tiys, Igor A. Popov, Natalia L. Starodubtseva, Igor V. Dobrokhotov, Vladimir A. Ivanisenko, Irina M. Larina, Nicolay A. Kolchanov, Evgeny N. Nikolaev

**Affiliations:** 1 Institute of Biomedical Problems – Russian Federation State Scientific Research Center RAS, Moscow, Russia; 2 Gagarin Cosmonauts Training Center, Star City, Russia; 3 Emanuel Institute of Biochemical Physics RAS, Moscow, Russia; 4 Institute for Energy Problems of Chemical Physics RAS, Moscow, Russia; 5 Moscow Institute of Physics and Technology, Moscow, Russia; 6 Institute of Cytology and Genetics SB RAS, Novosibirsk, Russia; 7 Research Center for Obstetrics, Gynecology, Moscow, Russia; 8 Orekhovich Institute of Biomedical Chemistry of the Russian Academy of Medical Sciences, Moscow, Russia; Moffitt Cancer Center, United States of America

## Abstract

The urine protein composition samples of ten Russian cosmonauts (male, aged of 35 up to 51) performed long flight missions and varied from 169 up to 199 days on the International Space Station (ISS) were analyzed. As a control group, urine samples of six back-up cosmonauts were analyzed. We used proteomic techniques to obtain data and contemporary bioinformatics approaches to perform the analysis. From the total number of identified proteins (238) in our data set, 129 were associated with a known tissue origin. Preflight samples contained 92 tissue-specific proteins, samples obtained on Day 1 after landing had 90 such proteins, while Day 7 samples offered 95 tissue-specific proteins. Analysis showed that consistently present proteins in urine (under physiological conditions and after space flight) are cubilin, epidermal growth factor, kallikrein-1, kininogen-1, megalin, osteopontin, vitamin K-dependent protein Z, uromodulin. Variably present proteins consists of: Na(+)/K(+) ATPase subunit gamma, β-defensin-1, dipeptidyl peptidase 4, maltasa-glucoamilasa, cadherin-like protein, neutral endopeptidase and vascular cell adhesion protein 1. And only three renal proteins were related to the space flight factors. They were not found in the pre-flight samples and in the back-up cosmonaut urine, but were found in the urine samples after space flight: AFAM (afamin), AMPE (aminopeptidase A) and AQP2 (aquaporin-2). This data related with physiological readaptation of water-salt balance. The proteomic analysis of urine samples in different phases of space missions with bioinformation approach to protein identification provides new data relative to biomechemical mechanism of kidney functioning after space flight.

## Introduction

The protein composition (proteome) of the human physiological fluids can change due to internal and environmental factors, thus reflecting the functional state and adaptive potential of the human system. Human proteome is characterized by plasticity, i.e. variability of the composition of components functioning at any specific time. The proteomic analysis of urine has certain technical advantages, such as sample stability due to the small amounts of proteases and relatively low concentrations of the major proteins – albumin and globulins [1]. Urinary proteome has been extensively studied in the last decade for its diagnostic, disease process monitoring and prognostic utility [2]. It is considered that proteomics achieved the best results in practical application of its findings specifically in the study of urine proteome [3].

It is notable that the attitude towards proteinuria in physiology has always been complicated. The development of quantitative protein detection methods disproved the long-standing belief that normal urine is protein-free; proteins are present in urine in very small concentrations that may increase in pathological conditions. Proteomic methods dramatically decreased the detection threshold and allowed accurate identification composition of the excreted proteins. Due to new analytic abilities there is an increased interest to study urine protein composition of healthy humans after space flight. This opportunity is of great interest to space medicine and physiology, since the exposure to the factors of space flight is known to affect water-salt balance and renal function [4]. We undertook a study of urinary protein composition to better understand the molecular mechanisms of homeostasis, which in turn might lead to improvements in the protection of crewmember health and well-being, especially in missions of extended duration (up to one year or longer). This work focuses on proteomic analysis of urine obtained from Russian cosmonauts - long duration crewmembers the International Space Station (ISS) and their back-ups. To our knowledge, urinary proteomic analyses with appropriate bioinformatics analysis have not been conducted in any astronaut populations.

## Materials and Methods

Urine samples from ten Russian cosmonauts (male) aged 35–51 years were analyzed. All subjects provided written informed consent to participate in the experiment «Proteome» in advance of their long duration (169–199 days) missions on the ISS. The experiment «Proteome» was approved by Biomedicine Ethics Committee of the Russian Federation Scientific Research Center-Institute of Biomedical Problems, Russian Academy of Sciences/Physiology Section of the Russian Bioethics Committee Russian Federation National Commission for UNESCO and Human Research Multilateral Review Board, NASA, Houston, Tx, USA. Urine samples were collected 30 days before launch and on the first and seventh days after landing, from the second morning fraction, which is known to the least variable fraction in terms of protein composition [5]. As a control group, urine samples of six back-up cosmonauts were analyzed. This group is epidemiologically identical to the cosmonaut cohort. Sex, age and protocol of investigation were the same to the prime cosmonauts group.

The specimen collection methodology (samples processing, time to storage, storage conditions etc.) was identical in all cases. Midstream of the second-morning urine was obtained, and the samples were stored immediately at 4°C. Urine samples were centrifuged at 2000 g for 10 minutes at 0–4°C to remove cell debris and the supernatant was frozen at −86°C until usage. Preparation of samples for mass spectrometric analysis followed a standardized procedure described earlier [6].

### Sample Preparation for Mass Spectrometry Analysis

Urine samples (15 mL) were concentrated using Amicon Ultra Ultracel-15 5 k tube (Millipore, USA) at 1000 g for 1 h at 4°C. The resultant concentrate (300 µl) was then evaporated to dryness in a centrifuge evaporator. Samples were normalized up to total protein concentration of 10 mg/mL using reduction buffer containing 0.2 M Tris-HCl, pH 8.5, 2.5 mM EDTA, 8 M urea. Urinary protein level was measured by standard method with Bradford Protein Kit (Bio-Rad) according to manufacture recommendations. To reduce cysteine residues the solution of urinary proteins was mixed with dithiothreitol (0.1 M final concentration) and incubated at 37°C. For alkylation of reduced SH-groups, the reaction mixture was cooled and mixed with small amount of concentrated aqueous solution of iodoacetamide up to its final concentration of 0.05 M. After incubation of the reaction mixture at room temperature for 15 min in darkness, the reaction was stopped by adding molar excess of 2-mercaptoethanol (10 µl per mg of added dithiothreitol). Proteins were precipitated by addition of 10 volumes of acetone containing 0.1% (v/v) trifluoroacetic acid and overnight incubation at –20°C. After centrifugation at 12000 g for 10 min at 4°C the sediment was resuspended in 96% ethanol (v/v), centrifuged again at 12000 rpm for 10 min at 4°C, and dried in the centrifuge evaporator for 1 h at 45°C. Trypsinolysis of the urinary protein fraction was performed in 200 mM NH_4_HCO_3_ buffer (protein concentration about 1 mg/mL) with modified porcine trypsin (Promega, USA) added at the ratio enzyme/protein of 1∶100 (w/w). After 6 h incubation at 37°C hydrolysis was stopped with formic acid (final concentration of 3.5%). The solution was centrifuged at 12000 g for 10 min at 4°C, and the supernatant was analyzed by chromatography–mass spectrometry.

### Liquid Chromatography-mass Spectrometry (LC-MS/MS)

LC-MS/MS experiments were performed in triplicate on a nano-HPLC Agilent 1100 system (Agilent Technologies, Santa Clara, CA, USA) in combination with a 7-Tesla LTQ-FT Ultra mass spectrometer (Thermo Electron, Bremen, Germany) equipped with a nanospray ion source (in-house system). A sample volume of 1 µl was loaded by autosampler onto a homemade capillary column (75 µl id × 12 cm, Reprosil-Pur Basic C18, 3 µm, 100 Å; Dr. Maisch HPLC GmbH, Ammerbuch-Entringen, Germany) which was prepared as described by Ishihama Y. *et al.* [7]. Separation was performed at a flow rate of 0.3 µl/min using 0.1% formic acid (v/v, solvent A) and acetonitrile 0.1% formic acid (v/v, solvent B). The column was pre-equilibrated with 3% (v/v) solvent B. Linear gradient from 3% to 50% (v/v) of solvent B in 90 min followed by isocratic elution (95%, v/v, of solvent B) for 15 min was used for peptide separation. MS/MS data were acquired in data-dependent mode using Xcalibur (Thermo Finnigan, San Jose, CA, USA) software. The precursor ion scan MS spectra (m/z 300–1600) were acquired in FT mode with a resolution of R = 50000 at m/z 400. The most intense ions were isolated and fragmented in LTQ.

### Data Analysis and Evaluation

LC-MS/MS data were searched using Mascot (Matrix Science, London, UK; version 2.0.04) against the human IPI protein sequence database from the European Bioinformatics Institute (version 3.82; released 06.04.2011; 92104 entries). Mass tolerance for protein identification was 5 ppm for MS and 0.50 Da for MS/MS. Proteins were considered to be correctly identified if more than two unique tryptic peptides were obtained with ion scores >24. Mascot results were filtered by software developed in Prof. Nikolaev’s group [6], [8]. All raw MS data and Mascot search results are submitted to the PeptideAtlas (submission PASS00239) repository and are freely available for download with the URL: http://www.peptideatlas.org/PASS/PASS00239. Identified proteins were annotated according to the information available from the UniProt KB, Tissue-specific Gene Expression and Regulation (TiGER), Gene Ontology.

## Results and Discussion

The results of the mass-spectrometric analysis of the urine samples were aggregated in a set of fifty-four (54) HTML files containing reports of IPI (International Protein Index) identification by the Mascot database. Files contained 430 different IPI indices with scores ranging from 20 to 1700. Proteome analysis due to its high sensitivity allowed evaluating the total urine protein composition. Each urine sample was analyzed three times by proteomic methods and the peptides discovered two or three times were selected for the further analysis. The information on attribution of IPIs to specific tissues was obtained from UniProtKB and Tissue-specific Gene Expression and Regulation (TiGER) databases. The latter contains information on tissue-specific gene expression for 30 different types of human tissue.

**Figure 1:**
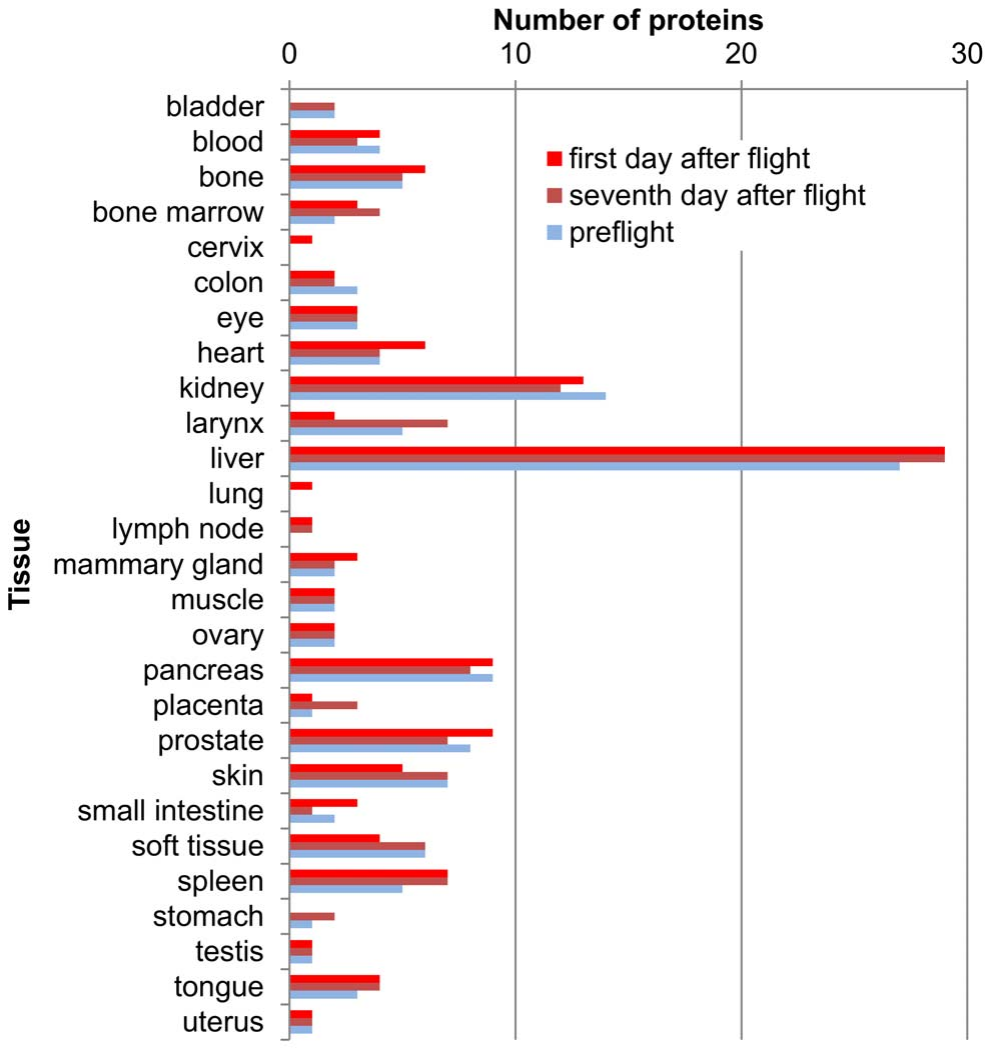
Distribution of specific tissue proteins. The number of proteins for each tissue accordingly to the TiGER database. *Preflight* condition is a condition of cosmonauts before the flight. The *first day after* flight is a landing day, and the *seventh day after flight* is a week after the landing. There are numbers of proteins form the united proteome of 10 cosmonauts.

**Figure 2:**
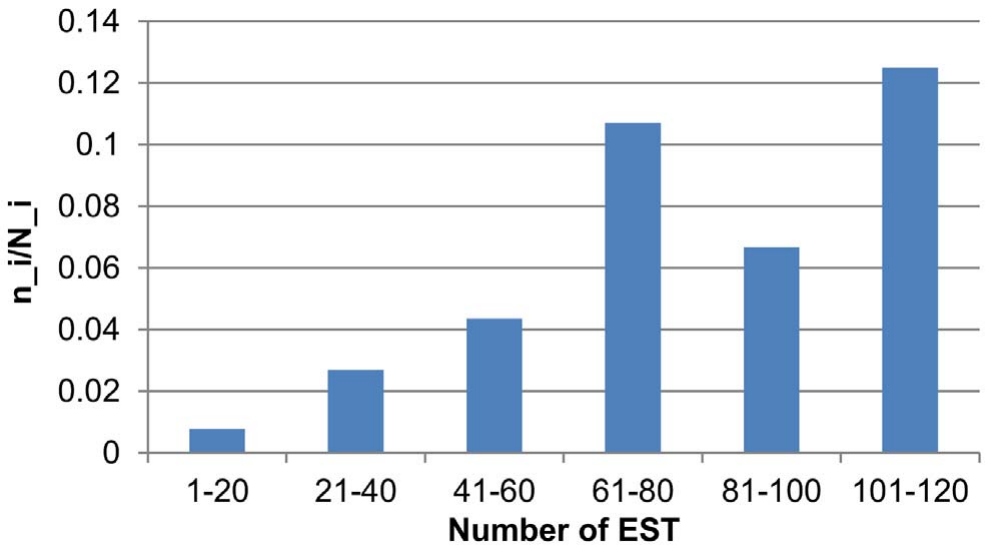
The portion of proteins presented in urine according to the number of EST. *N_i* is a number of proteins from TiGER database with *i* level of EST. n_i is a number of urine proteins with *i* level of EST, *i* is an interval of EST values. EST values serve as a rough estimation of gene expression.

From the total number of identified proteins (238) in our data set, 129 were associated with a known tissue origin **(** **Figure 1** **. Distribution of specific tissue proteins).** Preflight samples contained 92 tissue-specific proteins, samples obtained on Day 1 after landing had 90 such proteins, while Day 7 samples offered 95 tissue-specific proteins. Analysis of distribution of specific tissue proteins in each group of samples demonstrated that the protein spectra of the liver tissue, bones, soft tissue, kidney, prostate and pancreas had significantly stronger representation than the respective spectra of the corresponding tissues in the TiGER database (p<0.05 with consideration of Benjamini-Hochberg correction for multiple comparisons). Lymph node and testicular tissues were significantly less represented in all samples. Splenic tissue was significantly more represented only in the preflight samples. Increased or decreased levels (relative to TiGER database) of presence of the specific tissue proteins in urine revealed the complexity of penetration process of different proteins into the urine. One of the causes of this could be the difference in protein expression in diverse tissues leading to the different protein concentration entering the bloodstream, which, in its turn, reflects in the urinary protein spectrum. Indeed, our data show a reliable correlation between proteins represented in the urine and the EST (Expressed sequence tag) value of the corresponding genes in the tissue **(** **Figure 2** **. The portion of proteins presented in urine according to the number of EST).** In the control back-up group was found 81 proteins.

The protein identification of all 430 peptides (per UniProt) was followed by analysis of their functions and cellular localization using the Gene Ontology database. We thereby found that 24.5% of the identified proteins were involved in regulatory processes, 10.8% in organogenesis, 10% in regulatory responses, and 9.5% in cellular interaction. As to molecular functions, the majority (54%) were proteins involved in binding/transport of macromolecules and metabolites while 38.7% were regulators of cellular functions.

Out of the total number of detected proteins (430) in TiGER database, 23 IPIs were attributable to kidneys, i.e. these IPI were markers of proteins expressed in kidney. These are of utmost importance for the goal of the study since kidneys are the executive organ of homeostatic systems involved in maintenance of internal environment constants in the challenging conditions of long-duration microgravity and other major factors of space flight [4].

There were a total of 18 proteins originating from the renal tissue **(** **Table 1** **)**. Those were divided into three groups: consistently present (I), variably present (II) and proteins related to space flight (III).

**Table 1:** Renal proteins in the prime and back-up cosmonauts’ urine before and after space flight.

Renal tissue proteins	Back-up cosmonauts (n = 6)		Prime cosmonauts (n = 10)		
	Before flight	190–240 after	Before flight	1st day after landing	7th day after landing
CUBN, cubilin	present	present	present	present	present
EGF, epidermal growth factor	present	present	present	present	present
KLK1, kallikrein-1	present	present	present	present	present
KNG1, kininogen-1	present	present	present	present	present
LRP2, megalin	present	present	present	present	present
OSTP, osteopontin	present	present	present	present	preset
PROZ, vitamin K-dependent protein Z	present	present	present	present	present
UROM, uromodulin	present	present	present	present	present
ATNG, Na(+)/K(+) ATPase subunit gamma	absent	present	absent	absent	absent
BD01, β-defensin-1	absent	absent	present	present	present
DPP4, dipeptidyl peptidase 4	absent	present	present	absent	present
MGA, maltasa-glucoamilasa	absent	absent	present	present	absent
MUCDL, cadherin-like protein	absent	absent	present	absent	absent
NEP, neutral endopeptidase	absent	absent	present	absent	absent
VCAM1, vascular cell adhesion protein 1	absent	absent	present	present	present
AFAM, afamin	absent	absent	absent	**present**	absent
AMPE, aminopeptidase A	absent	absent	absent	absent	**present**
AQP2, aquaporin-2	absent	absent	absent	**present**	absent

Analysis showed that following group I proteins, consistently present in urine, have renal tissue as their source: CUBN (cubilin), EGF (epidermal growth factor), KLK1 (kallikrein-1), KNG1 (kininogen-1), LRP2 (megalin), OSTP (osteopontin), PROZ (vitamin K-dependent protein Z), UROM (uromodulin). Group II, variably present proteins, consists of: ATP1G1 (Na(+)/K(+) ATPase subunit gamma), BD01 (β-defensin-1), DPP4 (dipeptidyl peptidase 4 (CD26)), MGA (maltasa-glucoamilasa), MUCDL (cadherin-like protein), NEP (neutral endopeptidase) and VCAM1 (vascular cell adhesion protein 1). And only three renal proteins were related to the space flight factors. They were not found in the pre-flight samples and in the back-up cosmonaut urine, but were found in the urine samples after space flight (group III): AFAM (afamin), AMPE (aminopeptidase A) and AQP2 (aquaporin-2). The peptides identified for afamin, aminopeptidase A, and aquaporin 2 attached in Table 2. (**Table 2**).

**Table 2:** Peptides identified for the pos-flight proteins.

Number of detections	Protein	Peptides	Score	Mass accurace, ppm
1	Aquaporin2	SLAPAVVTGK	29	−0.13
		GLEPDTDWEER	67	0.79
2	Aminopeptidase A	ETNLLYDPK	41	−0.51
		ASLIDDAFALAR	64	0.07
		TSDFWAALEEASR	75	0.73
		TQDVFTVIR	56	−1.51
		ANPSQPPSDLGYTWNIPVK	44	1.58
		TSLAQEK	18	−2.85
2	Afamin	SDVGFLPPFPTLDPEEK	36	1.21
		HELTDEELQSLFTNFANVVDK	59	−0.48
		IAPQLSTEELVSLGEK	80	0.27
		FLVNLVK	44	−0.23
		HFQNLGK	25	2.22
		LPNNVLQEK	27	1.52

Further, place of synthesis and functions of these proteins in the different parts of nephron were analyzed.

Cubilin (CUBN, MB 398,736 Da) and megalin (LRP2, MB 521,958 Da) are membrane glycoprotein receptors. Megalin and cubilin are involved in receptor-mediated endocytosis in the apical membrane of proximal tubular epithelial cells [9]. They play a critical role in the reabsorption of glomerular-filtered proteins and they are elements of a low-affinity, high-capacity system. These multi-ligand receptors bind distinct but overlapping sets of ligands. It has been demonstrated that the megalin/cubilin-mediated reabsorption of vitamin D binding protein is responsible for the renal conversion of 25(OH)D3 to 1,25(OH)2 D3 in the proximal tubule. For transcobalamin and retinol-binding protein, the reabsorption appears to preserve vitamin B12 and vitamin A, respectively, for the organism [10]. Appearance in urine fragments of membrane receptors suggest that they are excreted through the process of exosome formation in proximal tubular epithelial cells.

The proteins of the kallikrein-kinin system kininogen-1 (KNG1, MB 71,957 Da) and kallikrein-1 (KLK1, MB 28,890 Da) turned out to be consistently present in urine as well. This is a key proteolytic system. The kallikrein–kinin system is composed of kallikrein, kininogen, kinin receptors, and kininase, kinin, and plays important roles in regulation of different physiological functions of human organism. KLK1 is synthesized in many organs, including kidneys and arteries, where it can generate the vasodilators bradykinin and kallidin. In kidney kallikreins are synthesized in proximal tubular epithelial cells, which are the source of their release into the urine [11].

Experimental and clinical studies have shown an inverse correlation between urinary kallikrein levels and blood pressure. KLK1 is likely involved in the maintenance of normal cardiac, renal, and neurological function [12]. It has been suggested that the kallikrein–kinin system can protect from hypoxia, prevent interstitial fibrosis, mediate vasodilatation and inflammation, and activate the innate immune system.

Epidermal growth factor (EGF, MB 133,994 Da) regulates cell proliferation through the IGF receptor. It has been shown that EGF stimulates magnesium reabsorption in the distal convoluted tubule [13].

Osteopontin (OSTP, MB 35,423 Da) is a highly phosphorylated glycoprotein present in many tissues and body fluids. In urine, OSTP is a potent inhibitor of nucleation, growth and aggregation of calcium oxalate crystal; this suggests a possible role in the prevention of renal stone formation. It has been identified among the major protein components of renal calculi [14], but its role in nephrolithiasis is somewhat unclear.

Vitamin K-dependent protein *Z* (PROZ, PZ, MB 44,744 Da) is a multi-domain vitamin K-dependent plasma protein. It functions in bones and arteries, regulating the activity of matrix Gla-protein (MGP) and osteocalcin (cBGP). cMGP inhibits vascular calcifications, while cBGP has an important role for a proper mineralization process [15]. These proteins play pivotal roles in the physiology of mineralization and in preventing ectopic calcification.

Uromodulin (Tamm-Horsfall protein, MB 69,761 Da) is the most abundant protein excreted in the urine under physiological conditions. Its biological functions have been linked to water/electrolyte balance and to kidney innate immunity. It is exclusively found in the thick ascending limb cells and early distal convoluted tubule of the nephron, where it is produced on apically targeted, eventually being secreted into the urine. Uromodulin is glycosylphosphatidylinositol-anchored protein. Uromodulin expression resulted in a significant increase of neutrophil adhesion (after binding the heavy and light chains of IgG) and trans-epithelial migration, in both the apical-to-basolateral and the basolateral-to-apical directions [16].

Thus, eight proteins consistently present in the urine with renal tissue as their source have regulatory functions, as well as they are membrane proteins and involved in reabsorption of proteins in the proximal tubular epithelial cells.

Seven proteins of renal tissue are characterized as variably present in cosmonaut’s urine (n = 16): vascular cell adhesion protein 1 (VCAM1 MB 81,276 Da), β-defensin-1 (BD01, MB 7,420 Da), dipeptidyl peptidase 4 (DPP4, MB 110 κDa), neutral endopeptidase (NEP, MB 85,514 Da), ATP1G1 (Na(+)/K(+) ATPase subunit gamma), MGA (maltasa-glucoamilasa) and MUCDL (cadherin-like protein).

The dynamic of presence in urine of AFAM, AMPE, and AQP2 was related to space flight.

Afamin (AFAM, MB 69,069 Da) is alpha-tocopherol binding protein. It plays an important role in protection from oxidative stress. It functions as an antioxidant and antiapoptotic protein [17]. Altered afamin urine concentration is observed in hypercalcemia with increased endothelium-dependent relaxations, mediated via Ca++ -activated K+-channels [18].

Glutamyl aminopeptidase (AMPE, also known as aminopeptidase A) is the principal vital enzyme which helps to regulate blood pressure through degradation of angiotensin II. Aminopeptidase A is apical membrane peptidase of proximal tubular epithelial cells. It intermittent appears in urine as a marker of mild and reversible tubular dysfunction [19]. The protein was revealed in the urine only on the seventh day after spaceflight; it was not present in the pre-flight samples or in the first day after flight samples.

**Figure 3:**
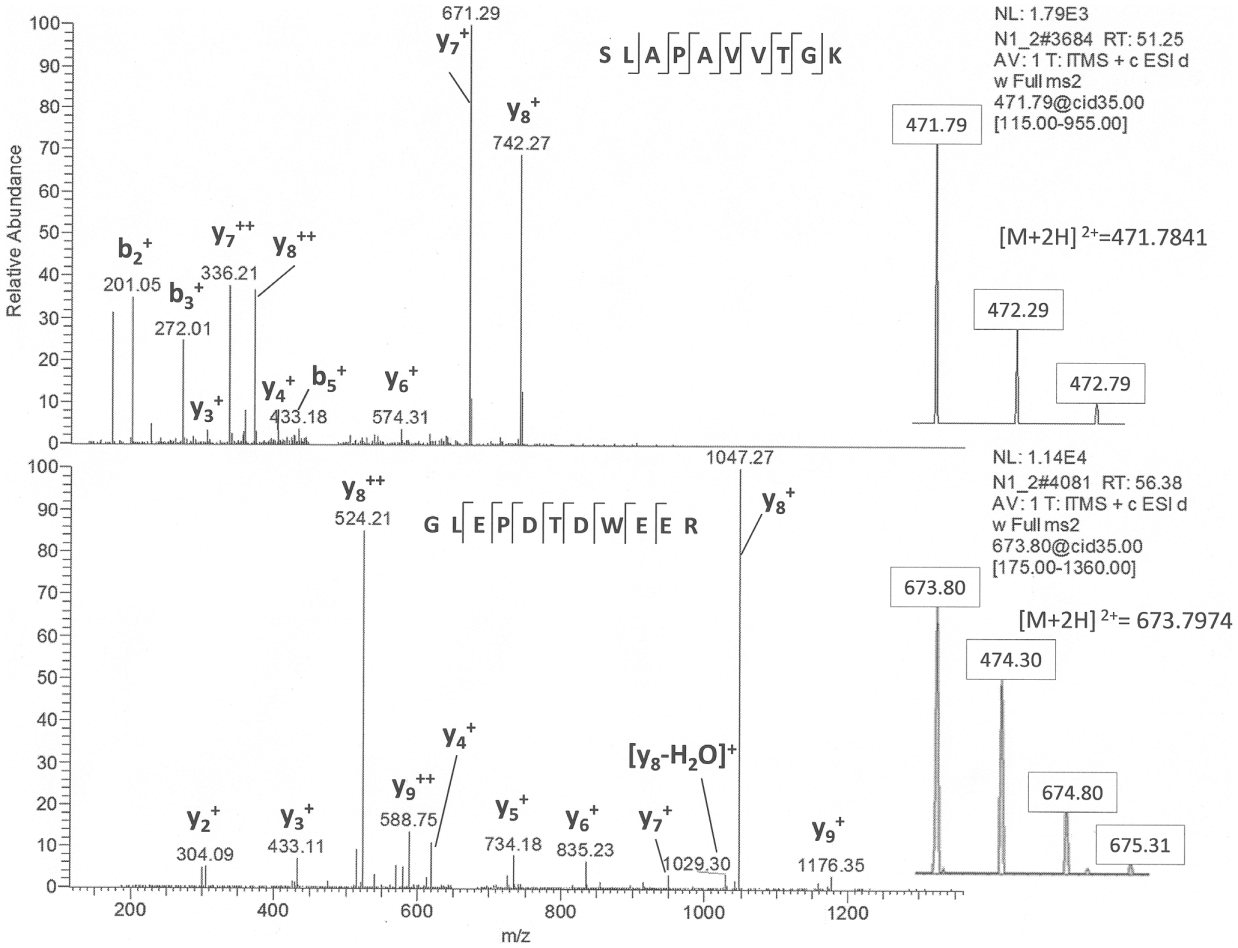
MSMS data for two peptides of aquaporin 2.

In the post-flight urine samples we revealed aquaporin-2 (AQP2). MSMS data for two peptides of aquaporin 2 is on the Figure 3 **(** **Figure 3** **. MSMS data for two peptides of aquaporin 2)**. AQP2 is an apical membrane protein of renal collecting duct epithelial cells that forms molecular channels (pores) for water transport across lipid membrane. Aquaporins selectively conduct water molecules while preventing the passage of charged ions. Renal water excretion is mainly regulated through effects of vasopressin on the aquaporin-2 (AQP2). Altered AQP2 protein presence in the renal collecting duct is largely responsible for water balance abnormalities associated with lithium-induced diabetes insipidus, congestive heart failure, and the syndrome of inappropriate antidiuresis. The excretion of aquaporin-2 in the urine increases in response to vasopressin [20]. Alteration in AQP2 trafficking and increased renal reabsorption of water results in AQP2 entering into the urine with calcineurin A alpha (CnAalpha).

Seemingly, exosomes can transfer functional AQP2 between cells and the amount of AQP2 in exosomes released from collecting duct cells is physiologically regulated. AQP2 excretion is significantly higher during high salt intake, indicating that water transport via AQP2 is increased.

Inhibition of prostaglandin synthesis decreased renal sodium excretion by increased absorption of sodium. Urinary AQP2 decreased indicating that water transport via AQP2 fell. Antidiuretic hormone and calcitonin induces cAMP-dependent AQP2 trafficking in cortical collecting and connecting tubules in parallel with an increase in urine concentration. Modeling of aquaporin-2 trafficking showed that phosphorylation of aquaporin-2 regulates its endocytosis and endocytosis [21].

The link between aquaporin 2, urinary concentrating function, hypercalcemia and hypercalciuria after space flight requires thorough analysis. Urinary calcium excretion during space flight is caused by bone reabsorption, which causes hypercalcemia, increased filtered load, and reduced tubular reabsorption [4], [22]. Urinary calcium increases early and tends to remain elevated throughout space flight, thus leading to a progressive negative calcium balance [22]. In the Skylab missions 2 through 4 urinary calcium excretion increased to a plateau and the magnitude of the in-flight increase were more than double the control preflight levels.

Studies on renal hemodynamic in space point to a moderate increase in glomerular filtration rate during the first 2 days after launch. Subsequently, renal hemodynamic reaches a new equilibrium that may not differ from conditions observed during head-out water immersion [23].

Fluid regulation and balance in space are different from that occurring on Earth. Under microgravity conditions occur a reduction of intravascular compartments associated with overflow in the interstitium. A positive sodium balance is therefore achieved, which was measured in 7 astronauts during SLS1 and SLS2 missions [24]. The renal response to water or saline load is impaired [25], [26]. Urinary flow rate after a 600 ml water load is lower than on Earth and independent of length of stay on orbit. Sodium balance is positive because renal sodium excretion is below intake. It was showed in the Skylab 3 mission, in the Apollo 17 mission and in the European Mir mission, where sodium intake averaged 178±11 mmol/day and urinary excretion averaged 121±11 mmol/day (P<0.002) [24].

It was hypothesized by Russian specialists that space flight might promote albuminuria [27]. However study aboard the Mir showed that a consistent reduction of microalbuminuria occurred compared with corresponding data on Earth [28] (**Table 3** **).**

**Table 3:** Microalbuminuria (mg/day) during Space Mission.

	Astronaut 1	Astronaut 2	Astronaut 3	Astronaut 4
Urinary albumin on Earth	3.24	6.13	4.47	3.90
Urinary albumin in space	2.54	4.18	3.01	3.90
Mean excretion on Earth	4.70±1.20			
Mean excretion in space	3.41±0.56(−27,4%), P<0.017			

Data from Cirillo et al. (28).

Only speculative explanations are possible. Either a direct effect of the various factors affecting urinary albumin excretion or an indirect effect mediated by redistribution of body fluid.

Vasopressin regulates the water permeability of the kidney collecting duct by trafficking aquaporin 2 from intracellular vesicles to the luminal plasma membrane. As a consequence water is reabsorbed, leading to urine concentration [29]. Urinary excretion of AQP2 is a potent marker for the diagnosis of water balance disorders. Compared with healthy subjects, the urinary excretion of AQP2 is very low in patients with central diabetes insipidus, in children with primary nocturnal enuresis, and conversely is much higher in patients with impaired water excretion [30]–[32].

Valenti et al. showed that hypercalciuria is associated with alterations of urinary AQP2 levels [33]. However, the factors that link hypercalciuria, associated polyuria, and AQP2 are unknown. Urinary concentrating defects and polyuria are the most important renal manifestations of hypercalcemia and the resulting hypercalciuria. It was proposed that a calcium-dependent calpain activation modulates AQP2 levels through AQP2 proteolysis [34].

The kidney is a key organ for calcium homeostasis. Its ability to sense extracellular calcium levels is attributable to the extracellular calcium sensing receptor, which is expressed in multiple sites along the nephron [35] and plays crucial role in the regulation of divalent mineral cation transport. Renal calcium sensing receptors are expressed on the apical membranes of proximal tubules, on the basolateral membranes of thick ascending limbs, and in a diffuse pattern in the distal convoluted tubules [36]. In the collecting duct, they are expressed in the apical membrane, where they are might sense urinary calcium levels and influence AQP2 trafficking, providing a link between calcium and water homeostasis [37].

Activation by high luminal calcium of the apical calcium sensing receptors in the terminal collecting duct, together with activation of calcium sensing receptors expressed in the thick ascending limb, causing a reduction in the countercurrent multiplication gradient might contribute to blunt water reabsorption and prevent further calcium concentration, protecting from a potential risk of nephrolithiasis.

During chronic adaptation of water balance to space is observed a reduction in plasma volume associated with low atrial natriuretic peptide concentrations and increased renin, norepinephrine, and vasopressin concentrations. A saline load and water load are excreted at a slower rate. The impairment in calcium metabolism might have a role in leading to AQP2 suppression. Thus water balance is kept constant (new level), and sodium balance increases continuously.

### Conclusion

To date, urinary proteome analysis has revealed from 1500 to 3500 different proteins [1], [2]. The specific functions of human cell populations and tissues are determined by the expression of specific spectra of proteins; each cell type synthesizes a set of proteins specific for the given tissue. Obviously, biomarkers for altered functions of kidney and urogenital system can be found in urinary proteome [2]. 34.9% of urinary proteins are also present in plasma and penetrate into the urine through glomerular filtration. The remaining 65.1% (up to 2000 proteins) of the urinary proteome are secreted by epithelial cells of the kidney and urogenital system or added to the urine by means of cell death or secretion of exosomes [3].

To our knowledge, this is the first study to characterize both permanent and variable parts of human urinary proteome in relation to space flight, thus examining the possibility of occult disturbance of the urogenital system in space flight. In some functional conditions, both quantity and spectrum of urinary proteins may change and include proteins beyond the common spectrum of “physiologic proteinuria” (albumin, Tamm–Horsfall protein, immunoglobulin light chains, hormones and enzymes). We identified several urinary proteins by their tryptic fragments, which, according to the TiGER database, are tissue-specific to kidneys and urogenital system. In specific functional conditions quantity and quality composition of urine proteins are varied. In tubular dysfunctions brush-border enzymes from the proximal tubules release into the urine; these enzymes include neutral endopeptidase, dipeptidyl aminopeptidase IV, α-glucosidase, leucine aminopeptidase, alkaline phosphatase, γ-glutamyltransferase, alanine aminopeptidase and N-acetyl-β-D-glucosaminidase.

In this investigation we can state that cosmonaut’s urine after space flight contains afamin, aminopeptidase A, and aquaporin 2 which are absent in the variable part of human proteome under physiological conditions.

Aminopeptidase A belongs to the group of brush border enzymes of the proximal renal tubules. This protein has been associated with tubular dysfunctions, which may be caused by intermittent renal hypoxia [38].

Aquaporin-2 and afamin presence in the urine of cosmonauts may be associated with the factors of space flight by several different possible mechanisms. The excretion of aquaporin-2 in the urine increases in response to vasopressin. Aquaporin-2 reaches the urine through the secretion of small exosomes without destruction of epithelial cells. Damaged intracellular recirculation of aquaporin [39] and increased renal water reabsorption [20] are associated with the loss of aquaporin-2. This very process is characteristic for kidney conditions after space flight [4]. There are several potential sources of afamin in the post-flight urine samples. Altered afamin urine concentration is observed in hipercalcemia [17]. Calcitonin has vasopressin-like action on the AQP2 trafficking in the collecting tubules [40]. Afamin effects contractility of smooth muscles of vessels and urinary bladder via Ca++ -activated K+-channels and could have originated from those sources [41].

In conclusion, the proteomic analysis of urine samples in different phases of space missions with bioinformation approach to protein identification provides new data relative to biomechemical mechanism of kidney functioning after space flight. Further research in this area appears to be warranted.
